# TCDD exposure alters fecal IgA concentrations in male and female mice

**DOI:** 10.1186/s40360-022-00563-9

**Published:** 2022-04-21

**Authors:** Christine L. Foxx, Madeline R. Nagy, Aspen E. King, Dreycey Albin, Gregory K. DeKrey

**Affiliations:** 1 School of Biological Sciences, College of Natural and Health Sciences, University of Northern Colorado, Greeley, CO 80639 USA; 2 Department of Computer Science, College of Engineering and Applied Science, University of Colorado, Boulder, 80309 CO USA

**Keywords:** Antibody, Immunoglobulin A, Mouse, Circadian, Diurnal, Aryl hydrocarbon receptor, Dioxin

## Abstract

**Background:**

Activation of the aryl hydrocarbon receptor (AhR) can alter diurnal rhythms including those for innate lymphoid cell numbers, cytokine and hormone levels, and feeding behaviors. Because immune responses and antibody levels are modulated by exposure to AhR agonists, we hypothesized that some of the variation previously reported for the effects of AhR activation on fecal secretory immunoglobulin A (sIgA) levels could be explained by dysregulation of the diurnal sIgA rhythm.

**Methods:**

C57Bl/6 J mice were exposed to peanut oil or 2,3,7,8-tetrachlorodibenzo-*p*-dioxin (TCDD, 10 or 40 μg/Kg) and fecal sIgA levels were determined in samples collected every 4 h over 4 days.

**Results:**

Fecal sIgA concentrations were not significantly different between light and dark phases of the photoperiod in either male or female mice, and there were no significant circadian rhythms observed, but TCDD exposure significantly altered both fecal mesor sIgA and serum IgA concentrations, in parallel, in male (increased) and female (biphasic) mice.

**Conclusions:**

AhR activation can contribute to the regulation of steady state IgA/sIgA concentrations.

**Supplementary Information:**

The online version contains supplementary material available at 10.1186/s40360-022-00563-9.

## Background

Immunoglobulin A (IgA) is the most abundant antibody isotype produced by B cells in the gut lamina propria, and IgA is the predominant isotype found in feces [38]. Export of IgA into the intestinal lumen is performed predominantly by mucosal epithelial cells. J chain-containing IgA multimers in the lamina propria are bound by poly-Ig receptors (pIgRs) on the basolateral side of epithelial cells. These IgA-J chain-pIgR complexes undergo transcytosis to the luminal side where proteolytic cleavage results in the release of secretory IgA (sIgA) into the intestinal lumen [34, 40].

sIgA acts in several ways to influence health. It plays a role in protection against mucosally-transmitted pathogens [1, 51, 58, 62, 67] and can influence the composition and function of commensal microbe populations in the gut [15, 24, 34, 47, 73]. In turn, gut microbiota can influence fecal sIgA concentrations and IgA production [17, 41, 64]. Moreover, components of food (e.g., peanuts, shellfish) can stimulate IgA production; sIgA may function to exclude those materials from the body and protect against development of allergies [59]. Like hormones, feeding behaviors, and metabolic processes, secretion and/or concentrations of fecal sIgA have been reported to vary with diurnal rhythmicity [6, 13, 25, 29, 35, 71].

The aryl hydrocarbon receptor (AhR) is a ligand-activated transcription factor of the Per-ARNT-Sim family of proteins that regulates cellular function through a canonical genomic pathway and several non-genomic pathways. The AhR is present in most cells of the body, but the level of expression can vary with cell type, location, and developmental state [16, 63]. In the absence of ligand binding, the AhR traffics in and out of the nucleus but exists primarily in the cytosol as a complex with chaperone proteins [12]. Ligand binding enhances retention of the AhR in the nucleus. Dimerization with the  AhR nuclear translocator (ARNT) protein allows AhR:ARNT to function as a DNA binding complex that targets dioxin response elements, which regulate the expression of multiple genes associated with metabolism, circadian rhythms, immune function, hormone production, development, and other processes [37, 60, 66, 74, 75]. The AhR can also regulate cellular functions through non-genomic mechanisms such as via a ligand-activated E3 ubiquitin ligase activity and by regulating a cytosolic Src kinase pathway [36, 44, 52].

The AhR can be activated by a large number of chemically diverse agonists including drugs (e.g., leflunomide), environmental chemicals (e.g., benzo(a)pyrene), tryptophan metabolites (e.g., kynurenine) or photodegradation products (e.g., 6-formylindolo(3,2-*b*)carbazole), lipoxin A_4_, metabolites of hemoglobin, as well as various plant-derived compounds that are commonly found in food and the microbially-derived metabolites of those compounds [43, 55]. AhR agonist activity has been demonstrated in human blood using reporter assays, and these studies showed that the levels of AhR agonists changed significantly due to changes in diet alone [8, 57]. The function of the AhR has been most widely studied using the ligand 2,3,7,8-tetrachlorodibenzo-*p*-dioxin (TCDD), a compound that has high AhR affinity and agonist activity [2, 60].

Activation of the AhR suppresses B cell activation and class switch recombination [31, 63, 68, 78]. These effects have been proposed as the reason for the widely reported suppression of systemic primary antibody responses after AhR activation in both male and female animals [14, 26, 52, 61]. Whereas antigen-specific serum immunoglobulin M (IgM) and immunoglobulin G (IgG) responses have been universally shown to be decreased by AhR activation, Warren et al. [72] found that blood levels of influenza-specific IgA were significantly increased after TCDD exposure in female mice. Interestingly, they also showed that influenza-specific sIgA levels were unchanged by TCDD exposure in bronchoalveolar lavage fluid. Another paradoxical observation was made by Chmill et al. [7], who found that ovalbumin-specific fecal sIgA concentrations were increased by TCDD exposure in female mice. The effects of AhR activation on total (steady state) sIgA concentrations have also been examined. Zhang et al. [77] found significantly elevated fecal sIgA levels in male mice exposed to 2,3,7,8-tetrachlorodibenzofuran (TCDF) within 7 days of oral treatment. Similarly, Benson and Shepherd [3] found significantly elevated fecal sIgA levels up to 13 days after exposure to TCDD in a mouse colitis model using both male and female mice. Interestingly, Culbreath et al. [9] showed that depletion of AhR agonists from feed could reduce fecal sIgA concentrations in mice. These findings suggest that blood IgA and/or secreted sIgA responses may be differentially impacted by AhR activation in comparison to IgM and IgG.

Activation of the AhR alters diurnal rhythms in a way that is consistent with inhibition of the circadian clock [65, 74, 75]. In the gut, AhR activation leads to altered rhythms for innate lymphoid cell numbers and cytokine production [18]. AhR activation also dysregulates diurnal feeding behaviors and blood rhythms for corticosterone, prolactin, thyroid hormone, and melatonin [23, 49, 50]. Because sIgA concentrations have been reported to vary with diurnal rhythmicity [29, 71], we hypothesized that some of the variation reported for the effects of AhR activation on fecal sIgA levels could be explained by dysregulation of the sIgA rhythm. Our study found that fecal sIgA concentrations were not significantly different between light and dark phases of the photoperiod for either male or female mice, and there was no significant circadian rhythmicity. However, TCDD exposure significantly altered fecal sIgA concentrations in a sex- and dose-dependent manner.

## Methods

### Ethics statement

This study was carried out in strict accordance with the recommendations in the Guide for the Care and Use of Laboratory Animals of the National Institutes of Health. The protocol was approved by the Institutional Animal Care and Use Committee of the University of Northern Colorado. All efforts were made to minimize suffering. This study is reported in accordance with ARRIVE 2.0 guidelines [46].

### Animal maintenance

C57Bl/6 J mice were maintained at the Animal Research Facility, University of Northern Colorado, on a standard 12-h light/dark (L/D) cycle with lights on at 0700 h Mountain Standard Time (Zeitgeber time, ZT0) and lights off at 1900 h Mountain Standard Time (ZT12). In Experiment 1 (diurnal rhythmicity), mice were housed individually for a period of approximately 5 days in polypropylene shoebox-style cages that had been modified such that approximately 80% of the floor space was comprised of a galvanized wire mesh (1/8th inch mesh size -- sufficient to allow fecal pellets to pass through). In Experiment 2 (*Leishmania* infection), animals were housed individually in Optimice® cages containing Tek-Fresh bedding (#7099, Envigo). All animals were provided with food (Rodent Diet 2016, Envigo) and deionized water ad libitum. Animals were euthanized with an overdose of CO_2_.

### Treatments and infections

**Experiment 1** (diurnal rhythmicity). TCDD was purchased from Cambridge Isotope Laboratories (Andover, MA) and solutions were prepared in peanut oil as described previously [11]. The concentration of TCDD was confirmed by gas chromatography using the method of [30]. Each animal (36 total, 18 of each sex, 6 per treatment group within each sex) was randomly assigned to a treatment group and was given peanut oil (vehicle) or TCDD (in peanut oil) at one of various doses by gavage (0.01 mL/g body weight). TCDD is a highly lipophilic compound that is poorly metabolized and which results in a half-life of approximately 8-11 days in C57BL/6 mice [39]. Thus, TCDD activation of the AhR in vivo is persistent.

**Experiment 2** (*Leishmania* infection). Each animal (18 total, 9 per treatment group) was randomly assigned to a treatment group and was given peanut oil or TCDD as in Experiment 1. *Leishmania major* (LV39, RHO/SU/59/P, Neal, or P strain) promastigotes were maintained by biweekly passage through C57Bl/6 mice followed by re-isolation from foot lesions on a rotator at room temperature in Schnieder’s Insect medium supplemented with 10% (v/v) heat-inactivated fetal bovine serum, 5 μg/mL hemin, 50 μg/mL gentamycin, 100 U/mL penicillin, 100 μg/mL streptomycin, 10 mM Hepes, 116 μg/mL arginine, 36 μg/mL asparagine, 110 μg/mL sodium pyruvate, and 292 μg/mL *L*-glutamine [56]. One day after vehicle or TCDD treatment, mice were anesthetized and then infected by subcutaneous injection of 50 μL phosphate-buffered saline (PBS) containing 1 × 10^6^ stationary-phase promastigotes into one rear foot pad.

### Feces and serum collection

**Experiment 1** (diurnal rhythmicity). Mice were acclimated to individual housing for 7 days, subsequently treated with vehicle or TCDD in the morning (approximately 0900 h), and 27 hours later each mouse was placed into a wire mesh-bottomed cage which was suspended above a piece of absorbent paper. At 1400 h on that day, a minimum of two fecal pellets were collected from the paper below each suspended cage and stored frozen (− 20 °C). A fresh piece of absorbent paper was then placed under each cage. In this way, feces were collected every 4 hours for 4 days with minimal interaction between animals and investigators. In the afternoon of the last day (1300 h - 1500 h), each animal was euthanized, and blood was collected. Serum was isolated and stored frozen (− 20 °C).

**Experiment 2** (*Leishmania* infection). *L. major*-infected mice were euthanized in the afternoon (1300 h - 1500 h) on day 21 after infection (day 22 after vehicle or TCDD treatment). Blood was collected immediately by cardiac puncture and placed into serum separator vials (BD Biosciences). Serum was isolated and stored frozen (− 20 °C). A volume of feces approximating two pellets was collected from the colon of each mouse and frozen prior to processing.

### Analysis of fecal and serum antibodies

Feces were thawed and extracted in PBS containing 1:100 protease inhibitor cocktail (P8340, Sigma-Aldrich Company) as described by Lycke et al. [33] Fecal extract was stored frozen (− 20 °C) prior to analysis. Antibody levels in dilutions of serum or fecal extract were determined by enzyme-linked immunosorbent assay (ELISA) using ELISA plates (Nunc MaxiSorp™ or equivalent) and the methods described previously [5, 33, 76]. Highly isotype-specific capture (unlabeled, #1040-01, Southern Biotech) and detection (biotinylated, #1040-08, Southern Biotech) goat anti-mouse antibodies were used with streptavidin-conjugated horseradish peroxidase (#443066, BD Biosciences), tetramethylbenzidine and hydrogen peroxide substrates (#5120-0047, Vector Laboratories), a SpectraMax 190 absorbance plate spectrophotometer (Molecular Devices), and SoftMax Pro software (ver. 5.4.1, Molecular Devices LLC). In Experiment 1 (diurnal rhythmicity), total IgA concentrations were estimated from standard curves generated using purified mouse monoclonal antibodies (#553476, mouse IgA-kappa, BD Biosciences). In Experiment 2 (*Leishmania* infection), the IgA level of each mouse was collected as the optical density value measured by the spectrophotometer.

### Statistical analysis

In experiments examining diurnal rhythmicity, feces were collected from each of six animals per treatment group every 4 hours over 4 days. Fecal samples collected at the same time each day were considered replicates in accordance with guidelines set forth by Refinetti et al. [53] for assessing baseline diurnal rhythm presence/absence in the control group. To facilitate the analysis of the control and TCDD-exposed groups in the same statistical model, we expanded this replicate sampling approach to all mice in the study. These replicate values were averaged resulting in a sample size of *n* = 6 at each time point. Statistics regarding rhythmicity were calculated using the Cosinor program [53]. Other statistical analyses to identify differences between treatment groups or sample types using mixed analyses of variance were performed using SAS (ver. 9.4, SAS Institute Inc.) and graphically depicted using SigmaPlot (ver. 14, Systat Software). Hypothesis testing was performed by analysis of variance combined with post hoc all-pairwise *t*-tests to identify means that were significantly different (*p* < 0.05) using a stringency no lower than the Tukey test.

## Results

### Experiment 1: sIgA and IgA in female and male mice after TCDD exposure

**Fig. 1:**
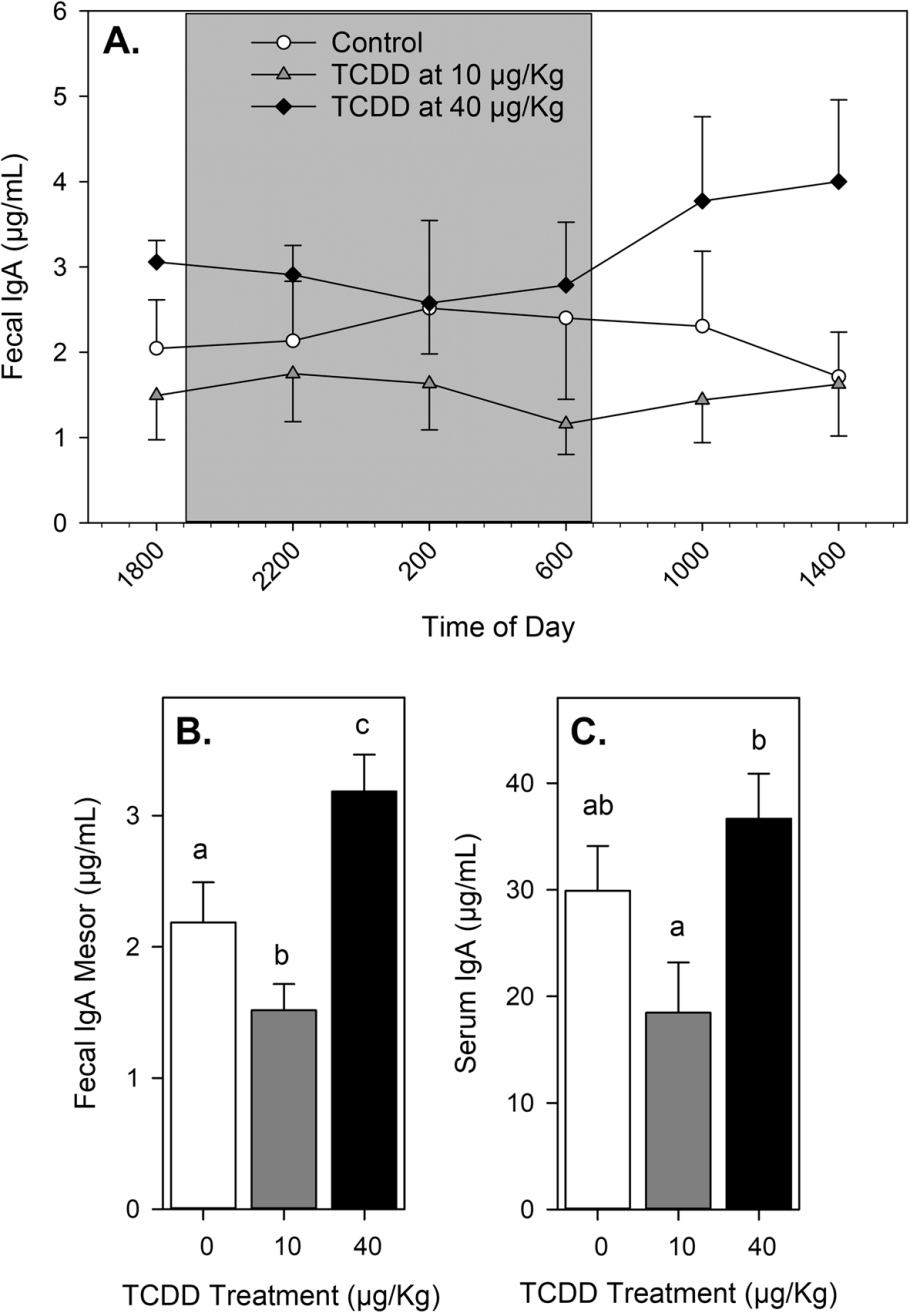
sIgA and IgA levels after TCDD exposure in female mice. Mice were given vehicle or TCDD (*n *= 6 per treatment group), rested for 1 day, and then feces were collected at 4-hour intervals for 4 days as described in the Methods. **A** Mean fecal IgA levels (+ and/or – SEM) at each daily time-point. **B** Mesor fecal IgA values (+ SEM). **C** Mean serum IgA levels (+ SEM) on day 5. Different letters indicate means that are significantly different at the same time-point (*p* < 0.05). Abbreviations: immunoglobulin A, IgA; standard error of the mean, SEM; 2,3,7,8-tetrachlorodibenzo-*p*-dioxin, TCDD

**Fig. 2:**
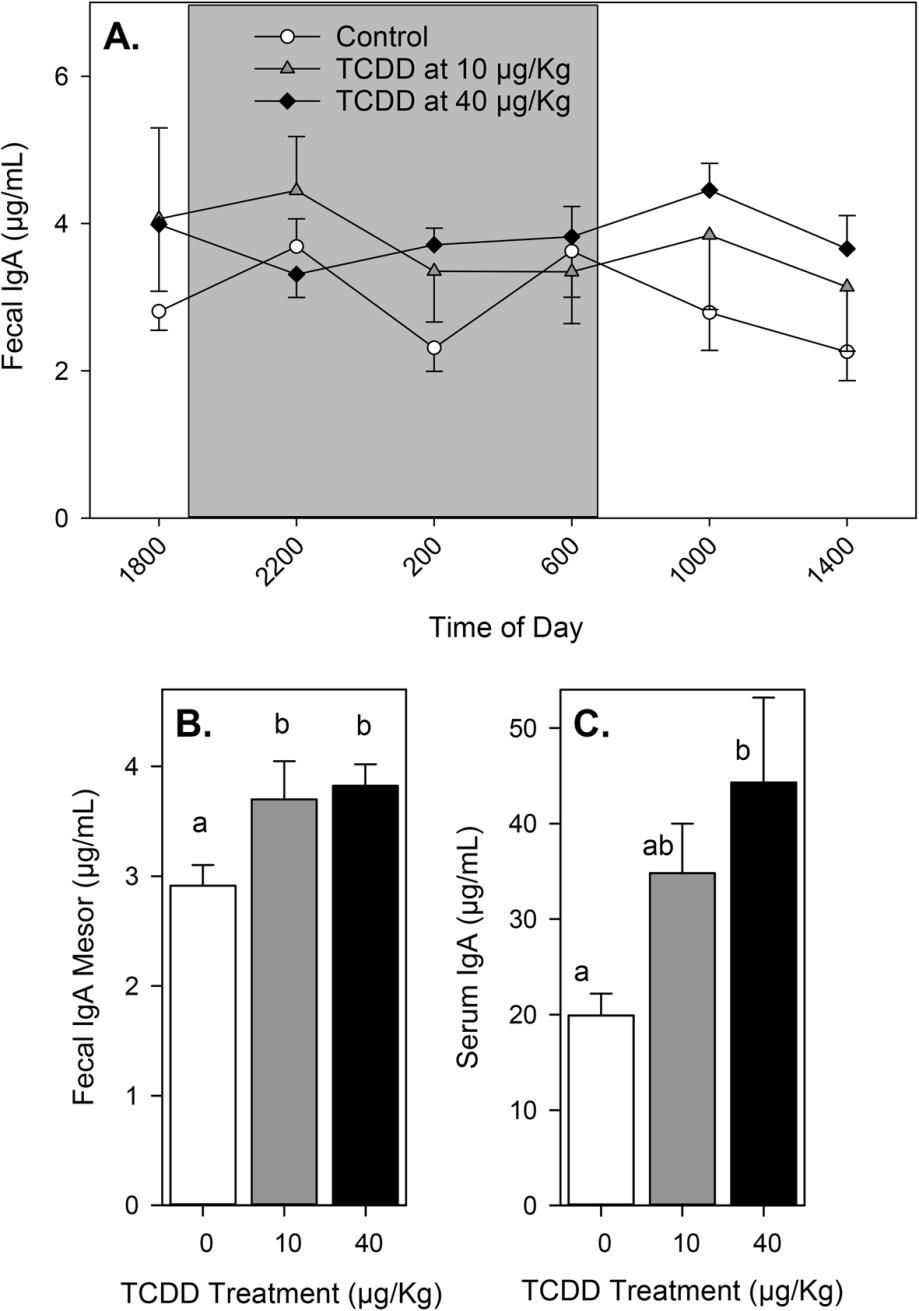
sIgA and IgA levels after TCDD exposure in male mice. Mice were given vehicle or TCDD (*n *= 6 per treatment group), rested for 1 day, and then feces were collected at 4-hour intervals for 4 days as described in the Methods. **A** Mean fecal IgA levels (+ and/or – SEM) at each daily time-point. **B** Mesor fecal IgA values (+ SEM). **C** Mean serum IgA levels (+ SEM) on day 5. Different letters indicate means that are significantly different at the same time-point (*p* < 0.05). Abbreviations: immunoglobulin A, IgA; standard error of the mean, SEM; 2,3,7,8-tetrachlorodibenzo-*p*-dioxin, TCDD

Beginning 1 day after vehicle or TCDD treatment, feces were collected every 4 hours for 4 days and analyzed for sIgA concentration. IgA levels in serum were determined at the end of the 4-day period. As shown in Figs. 1A and 2A, the highest sIgA levels in the feces of vehicle-treated control mice, (both female and male) were during the dark phase of the daily photoperiod. However, there were no significant differences between the mean values for the light and dark phases of either sex (Table 1). In addition, no significant circadian rhythmicity for fecal sIgA values in vehicle-treated animals of either sex was detected (Table 2). Among TCDD-treated animals at either dose (10 or 40 μg/Kg body weight), there were similarly no significant differences between sIgA mean values for the light and dark phases in either sex, and there was no significant circadian sIgA rhythmicity identified in either sex (Tables 1 & 2). However, when the - mesor values (arithmetic mean of all measured values) were calculated for female mice (Fig. 1B), a significant decrease of sIgA levels compared to vehicle-treated mice was observed at a moderate dose of 10 μg/Kg TCDD, whereas a significant increase compared to vehicle-treated mice was observed at a high dose of 40 μg/Kg. A different dose-response was observed in male mice (Fig. 2B), such that significantly higher fecal sIgA mesor values were observed in males treated with either the 10 or 40 μg/Kg TCDD dose compared to vehicle-treated controls. Serum IgA levels paralleled fecal mesor values in both female and male mice (Figs. 1C & 2C). Females given the lower TCDD dose had significantly lower serum IgA levels relative to controls, whereas females in the high dose group had significantly higher serum IgA levels relative to controls. Male mice contained higher serum IgA levels if given the low TCDD dose (not significant), and males in the high TCDD dose group showed significantly higher serum IgA levels.

**Table 1:** Fecal sIgA levels during light and dark periods^a^

Sex	TCDD (μg/Kg)	sIgA in Light Period (μg/mL)	sIgA in Dark Period (μg/mL)
Female	0	2.02 ± 0.37	2.35 ± 0.49
	10	1.52 ± 0.30	1.51 ± 0.28
	40	3.61 ± 0.45	2.76 ± 0.32
Male	0	2.62 ± 0.23	3.21 ± 0.29
	10	3.68 ± 0.58	3.72 ± 0.41
	40	4.03 ± 0.35	3.62 ± 0.18

^a^ Data represent means ± SEM for sIgA in fecal extract at 1000 h, 1400 h and 1800 h (light period), or 2200 h, 0200 h and 0600 h (dark period)

**Table 2:** Statistical analysis of fecal sIgA circadian rhythmicity^a^

Sex	TCDD (μg/Kg)	Robustness (%)	Values of P^b^
Female	0	11.8 ± 0.9	0.29 ± 0.03
	10	9.1 ± 3.2	0.44 ± 0.15
	40	19.3 ± 3.6	0.17 ± 0.06
Male	0	16.1 ± 3.5	0.24 ± 0.10
	10	20.0 ± 7.0	0.31 ± 0.12
	40	15.8 ± 3.9	0.21 ± 0.06

^a^ Data represent means ± SEM for *n *= 6 mice per treatment group sampled at 4-hour intervals for 4 days. Statistics were calculated using the Cosinor program [53]

^b^ Results of F tests to determine if the amplitude of modeled cosine waves is significantly larger than zero, an indication that the cosine fit is meaningful [53]

### Experiment 2: sIgA and IgA in *Leishmania*-infected female mice after TCDD exposure

**Fig. 3:**
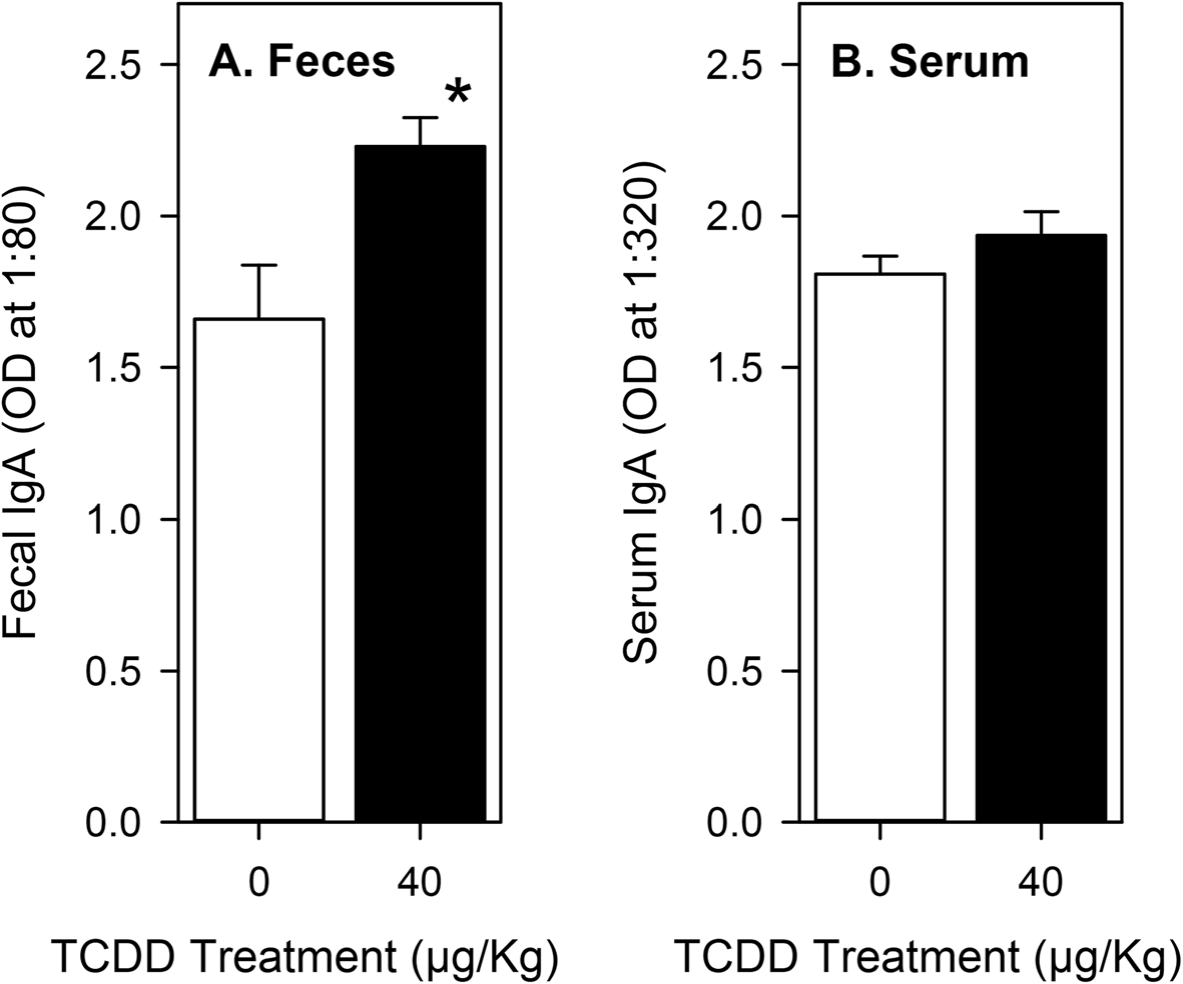
sIgA and IgA levels after 3 weeks in female mice. Mice were given vehicle or TCDD (*n *= 9 per treatment group) and then infected with *L. major* the following day as described in Methods. Feces and serum were collected 21 days after infection. The mean optical density (OD) values for IgA (+ SEM) are shown for **A** feces and **B** serum. * Indicate a mean that is significantly different from peanut oil controls (0 μg/Kg TCDD, *p* < 0.05). Abbreviations: immunoglobulin A, IgA; *Leishmania major, L. major*; optical density, OD; standard error of the mean, SEM; 2,3,7,8-tetrachlorodibenzo-*p*-dioxin

We reported previously [5] that a single dose of TCDD (40 μg/Kg) given to female C57Bl/6 1 day prior to foot pad infection with *L. major* resulted in over 60% suppression of serum *L. major*-specific antibody levels (all isotypes) 3 weeks after infection. In this current study, using the same treatment and infection regimen, we were unable to detect *L. major*-specific sIgA in the feces of control or TCDD-treated female mice when sample collections were made in the early afternoon (1300 h - 1500 h). However, we did find that total fecal sIgA and total serum IgA levels were readily detected. As shown in Fig. 3A, exposure to TCDD at a high dose (40 μg/Kg) resulted in a significant increase of total fecal sIgA levels 3 weeks after exposure by approximately 1.6-fold, relative to control animals. In contrast, TCDD exposure had no significant impact on total serum IgA levels (Fig. 3B).

## Discussion

To examine the diurnal variation of total (steady state) fecal sIgA levels in mice, we collected fecal pellets from animals of each sex every 4 hours over 4 days. In control (vehicle-treated) mice, we found no significant diurnal variation of fecal sIgA concentrations in either sex (Figs. 1A & 2A, Tables 1 & 2). These results contrasted with those from previous studies that suggested sIgA diurnal rhythms are present in saliva and feces of both male and female mice, with peak sIgA concentrations found during the light phase [6, 29, 71]. It should be noted that these previous studies did not collect samples repeatedly from the same animals over multiple days as recommended for modeling rhythmicity [53]. Given that the total fecal mass produced by rodents increases during the dark phase when they are most active [[13]; C. Foxx, personal observation], the concentration of sIgA in feces may not be reflective of the amount excreted during different times of the day. Indeed, when sIgA concentrations and fecal masses were used to calculate sIgA excretion rates in rats, peak excretion was found to occur during the dark phase [13, 25, 48, 54]. For this reason, the total fecal sIgA excreted by mice in our study most likely occurred during the dark phase.

The only significant effects of TCDD exposure on fecal sIgA concentrations in Experiment 1 of this study were for mesor values which changed in a dose- and sex-dependent manner (Figs. 1B & 2B). These results were paralleled by similar significant changes in serum IgA concentrations in both sexes (Figs. 1C & 2C). As discussed in Background, sIgA has been shown to provide protection against some mucosally-transmitted pathogens, to regulate gut microbe populations, and to potentially protect against development of allergies [1, 15, 24, 34, 47, 51, 58, 59, 62, 67, 73]. Extrapolation of our results might suggest that AhR activation could either inhibit or enhance resistance to intestinal pathogens and tolerance to allergens, depending on the agonist and its dose. However, because of the complexity of immune regulation and disease resistance, experimental evidence obtained using disease models would be more appropriate to explore such possibilities. Recent studies demonstrate that gut microbial community dynamics are influenced by the expression and activation of the AhR [42, 77]. Because members of the gut microbiota can influence fecal sIgA concentrations and IgA production [17, 41, 64], one explanation for the altered sIgA levels seen here in this study could be indirect regulation due to altered gut microbe populations. Alternatively, because sIgA can influence the composition and function of both pathogenic and commensal microbe populations [15, 24, 34, 47, 73], altered sIgA concentrations may be responsible, at least in part, for AhR-mediated changes in gut microbe diversity.

The impact of AhR activation on total fecal sIgA concentrations was examined previously by three different research groups. Kinoshita et al. [28] were the first to report a significant alteration, and they found that TCDD exposure (up to 20 μg/Kg body weight) dose-dependently decreased fecal sIgA concentrations in female C57Bl/6 mice, an effect that persisted for up to 3 weeks. Interestingly, Benson and Shepherd [3] found significantly elevated fecal sIgA levels up to 13 days after exposure to TCDD (30 μg total dose) in a mouse colitis model using both male and female C57Bl/6 mice. Similarly, Zhang et al. [77] found significantly elevated fecal sIgA levels within 7 days of treatment in male C57Bl/6 mice exposed to TCDF (24 μg/Kg total dose) or 2.4 μg/Kg TEQ [4]). For female mice, the results of our study are consistent with each of these previous studies, and collectively these results suggest that sIgA levels can be both reduced and increased by AhR activation, with the specific effect being dependent upon the dose of the AhR agonist as follows: 1) low to moderate doses of TCDD (≤ 20 μg/Kg) suppress fecal sIgA concentrations ([28], and Fig. 1B), but 2) high doses of TCDD (≥ 30 μg/Kg) enhance fecal sIgA concentrations ([3], and Fig. 1B). In male mice, the results are less clear. Ishikawa et al. [22] found that male pups exposed to TCDD through nursing had significantly lower sIgA in their feces (the mothers were exposed to an undisclosed “low dose” of TCDD, possibly 1 μg/Kg). In contrast, adult male mice given an AhR activating dose of 2.4 μg/Kg TEQ or higher experienced elevated sIgA levels within 2 weeks ([3, 77]; Figs. 1B & 2B). These results suggest that male mice may also experience a dose-dependent dichotomous impact on total fecal sIgA concentrations after AhR activation. However, the data also suggest that male mice may display a greater sensitivity than female mice to AhR activation, and the threshold for transition from suppression to enhancement is less definitive. It must be acknowledged that the only antibody isotype we examined in feces was IgA, and the possibility exists that other fecal antibody isotypes may be similarly impacted by TCDD exposure.

Data from several studies suggest that the sensitivity of mice to AhR-mediated immune regulation can depend on the sex of the animal [20, 27, 70]. One study examined a potential mechanism for this sex-dependent variation [10]. In that study, a greater sensitivity of male C57Bl/6 mice to suppression of an allogeneic graft rejection response was observed after AhR activation with 3,4,5,3′4’5′-hexachlorobiphenyl (HxCB, 10 mg/Kg). They found that castrated male mice were no more sensitive to HxCB-induced immune suppression than female mice. The authors suggested that AhR activation may increase the sensitivity of the immune system to testes-derived factor(s), and/or that HxCB treatment alters the production of testes-specific immunomodulatory factor(s). Given that blood testosterone levels were significantly reduced by HxCB-treatment in sham-castrated male mice, testosterone is not a likely contributor to altered immunity in this model. The potential effect of HxCB exposure on other hormones and testes-derived immunomodulatory factors remain to be seen, and further exploration of the mechanisms underlying AhR-sex interactions is warranted.

AhR activation causes suppression of naive B cell activation and suppression of class switch recombination from IgM to IgG and IgA [68, 78]. Therefore, a reasonable expectation could be to find significantly lower concentrations of serum antibodies in mice after TCDD exposure. This has been the exclusive observation after AhR activation for non-IgA total antibody levels using multiple models [45, 79] as well as for total IgA levels under some AhR agonist exposure regimens as discussed above. It has also been the exclusive observation for antigen-specific non-IgA isotypes after antigen challenge [19, 21, 32]. However, for antigen-specific IgA responses, the opposite appears to occur. Using a female mouse influenza infection model, Warren et al. and Vorderstrasse et al. [69, 72] independently found that TCDD exposure significantly elevated influenza-specific blood IgA levels at doses of 10 μg/Kg and lower (lower than those shown here) to elevate total IgA concentrations. Given that per cell antibody production has been found to be unaltered by AhR activation [78], a clear mechanistic explanation for increasing either blood or fecal IgA levels is lacking.

## Conclusions

AhR activation can contribute to the regulation of steady-state IgA/sIgA concentrations.

## Supplementary Information

**Additional file 1.**

## Data Availability

The datasets used and/or analyzed during the current study are included as supplemental materials.

## Abbreviations

AhR: Aryl hydrocarbon receptor
ARNT: AhR nuclear translocator
ELISA: Enzyme-linked immunosorbent assay
HxCB: 3,4,5,3′4’5′-hexachlorobiphenyl
IgA: Immunoglobulin A
IgG: Immunoglobulin G
IgM: Immunoglobulin M
L/D: Light/dark
PBS: Phosphate-buffered saline
pIgR: Poly-Ig receptor
sIgA: Secretory IgA
TCDD: 2,3,7,8-tetrachlorodibenzo-*p*-dioxin
TCDF: 2,3,7,8-tetrachlorodibenzofuran
ZT: Zeitgeber time
